# Assessment of Aflatoxins and Heavy Metals in *Zingiber officinale* (Ginger) Across the Value Chain in Kaduna and Oyo States, Nigeria and the Use of Probiotics for Aflatoxin Detoxification

**DOI:** 10.3390/foods15152620

**Published:** 2026-07-27

**Authors:** Titilayo D. O. Falade, Iyanu Lydia Ibrahim, Kolawole Banwo, Valery Pierre Sinda-Kemdoum, Rousseau Djouaka

**Affiliations:** 1 International Institute of Tropical Agriculture, Ibadan 200001, Oyo State, Nigeria; 2 Department of Microbiology, University of Ibadan, Ibadan 200001, Oyo State, Nigeria; 3 International Institute of Tropical Agriculture, Cotonou 0932, Benin; 4 International Institute of Tropical Agriculture, Accra P.O. Box M32, Ghana

**Keywords:** ginger, aflatoxins, heavy metals, risk assessment, probable daily intake, probiotics

## Abstract

*Zingiber officinale* (ginger) is a high-value crop with significant nutritional benefits, but it is susceptible to food hazards, including mycotoxins and heavy metals. Fifty-one ginger samples were collected along the value chain in Kaduna State (local government areas: Jaba, Kachia and Zango) and 44 samples obtained from markets in Oyo State (local government areas: Ibadan North, Ibadan Northeast, Ibadan Northwest, and Ibadan Southwest). The incidence of aflatoxins (B1, B2, G1, and G2) and heavy metals (mercury, cadmium, lead, copper, zinc, and chromium), as well as risk assessments, were determined. Half (50%) of the samples from Kaduna contained aflatoxins and all (100%) of the samples from Oyo State contained aflatoxins. On average, 18 to 60% of split-dried samples and 17 to 83% of fresh from Kaduna exceeded 10 ng/g total aflatoxin concentration. In Oyo State 67 to 100% of the split-dried samples exceeded 10 ng/g and 100% of both fresh and milled samples exceeded 10 ng/g total aflatoxins. The estimated probable daily intake of mercury (0.572 mg/kg bw/day) exceeded the limit of 0.23 mg/kg bw/day from Kaduna samples, whereas others were within the tolerable limits. Lead, which has no defined tolerable daily intake, was not detected in Kaduna State samples. In Oyo State samples, lead was detectable with an estimated limit of 0.007 mg/kg bw/day; whereas, the others were within the limit. The estimated probable daily intake (PDI) of aflatoxin B1 was estimated at 4.1 μg/kg bw/day and 1.5 μg/kg bw/day from Oyo State and Kaduna State samples, respectively. The provisional maximum tolerable daily intake (TDI) for aflatoxins is 2 μg/kg bw/day. The use of probiotics was explored for the detoxification of aflatoxin-spiked ginger samples (at 50 ng/g). *Lactiplantibacillus plantarum* ATCC 8014 (LP) and *Lactobacillus helveticus* ATCC 15009 (LH) reduced aflatoxin concentrations by 50% when used alone or together at the end of a 120 h incubation period. The study highlights the incidence and risk of exposure to multiple toxicants in ginger and the need for management of aflatoxins and heavy metals along the ginger value chain.

## 1. Introduction

*Zingiber officinale* Roscoe, commonly known as ginger, is a monocotyledonous crop of the Zingiberaceae family. Its rhizome is valued globally and is used majorly to flavour foods and beverages, it is also valued for its medicinal properties, e.g., anti-inflammatory, antioxidant properties, and for the prevention of neurodegenerative diseases [1,2,3]. In West Africa, fermented foods, drinks and cereal-based gruels (*zobo*, *kunun zaki*, *ogi* and *koko*) include ginger as an adjuvant for flavour [4,5]. Ginger is rich in vitamins (B6 and C), minerals (potassium, magnesium and manganese, phytochemicals (gingerol, shogaol) and, fibre (that aids digestion) [6,7]. These nutrients are important and contribute to normal body functioning, physiology and biochemical processes. Consequently, ginger is an important crop for healthy diets and for food security. Given its widespread use, the contributions of this crop to food security is critical.

In Africa, Nigeria is the largest producer and exporter of ginger. It is the second largest exporter of ginger globally after India, with 86,000 tonnes of ginger (valued at 475 million USD) [8]. However, losses from a disease outbreak in 2023 resulted in a decline in Nigeria’s share of the export market [9,10]. Nigerian ginger (yellow variety UG1) is referred to as *Tafingiwa* (translated as elephant foot) and the black variety UG2 is known as *Yatsunbiri* (translated as monkey finger) in Hausa [11,12]. The ginger varieties are sourced globally for their unique flavour and taste, with 80% of that produced in Nigeria being exported [11,12,13]. However, the ginger value chain is faced with challenges that threaten the quality and safety of the product from the country. Mycotoxin contamination has been reported in ginger samples including the varieties from Nigeria, in multiple academic literature [14,15,16,17]. Dietary exposure to heavy metals can be hazardous to health, especially when levels exceed the tolerable/safe limits [18,19,20]. However, there is limited information about the exposure of ginger to multiple food hazards along the value chain. As Nigeria has a high ginger market share, the better-quality ginger likely meets the regulatory requirements of importing countries (the majority of which are outside the African continent such as Asia, Europe and the United States of America) [8], as sub-optimal quality products will be rejected [21,22]. Consequently, this increases the burden of unsafe food in the domestic markets where monitoring measures are sub-optimal.

Mycotoxins are fungal toxins produced as secondary metabolites under the ideal conditions of crop stress (pre-harvest) and in enabling environments of saturation deficits, warm temperatures, and insect damage (post-harvest) [23,24,25]. Dietary exposure to mycotoxins has negative health impacts especially when ingested at elevated concentrations and outbreaks have been recorded [26,27]. Although there are no ‘safe’ concentrations for class 1 carcinogens like aflatoxins [18], regulatory limits are set at 10 ng/g for total aflatoxin concentration in ginger by the European Union commission [28]. High exposures to aflatoxin contamination have been linked to liver cancers, growth deficiencies, immune suppression, liver and kidney diseases [29,30,31]. It is therefore important to control the prevalence of this mycotoxin in crops and limit dietary exposure to consumers.

Within the production section of the value chain, in a temperate region, it has been reported that summer temperature (in contrast to winter) results in higher concentrations of aflatoxin [32]. Being a geocarpic crop, the rhizomes are also in direct contact with the soil that harbours a myriad of fungi, including *Aspergillus* section *Flavi*, which includes species and strains that are aflatoxigenic [33,34]. Other sections of the value chain, including processing (e.g., drying and packaging), can have an impact on the incidence of aflatoxins in crops [35,36,37].

Another food safety attribute of importance in ginger is heavy metal contamination [38]. Information about heavy metals across the ginger value chain in Nigeria is sparse. Heavy metals occur naturally in soils and it is possible for them to uptake into the crop, including the rhizome, during production [39,40]. Other routes of contamination could be practices which involve putting the crop in direct contact with contaminated soils, such as during sun drying on bare surfaces, storage in contaminated containers, or during transportation under sub-optimal conditions, all of which are avenues through which heavy metal contamination can occur. Heavy metals like mercury, species of arsenic, lead, chromium and copper are tightly regulated in food due to their negative impacts on human health, causing neurological defects and allergic reactions [41,42,43], and having associations with cancer (lead, cadmium, chromium) [44,45], infantile diseases and mortality (lead, mercury, arsenic, cadmium) [46,47].

Due to natural circumstances, it may sometimes not be possible to exclude contaminants in food completely. For instance, warm, humid regions of the world are prone to mycotoxin contamination due to the enabling climate conditions. Consequently, management methods that include decontamination are important. Probiotics with toxin-binding properties have been explored for decontamination, in addition to their benefits of improving gut microbiota, immune system boost, flavour, and aroma enhancement [28,48,49]. Lactic acid bacteria have been beneficial in aflatoxin reduction of up to 96.58% due to their toxin-binding abilities [50,51].

The current study investigated the incidence of mycotoxins (aflatoxins) and selected heavy metals in ginger samples collected from along the value chain in Nigeria, including production, drying, storage, milled, and traded ginger in the domestic informal markets. Additionally, the potential of probiotic strains was examined for decontamination of contaminated fresh ginger aflatoxin-spiked samples.

## 2. Materials and Methods

### 2.1. Sampling Sites and Sample Types

**Figure 1:**
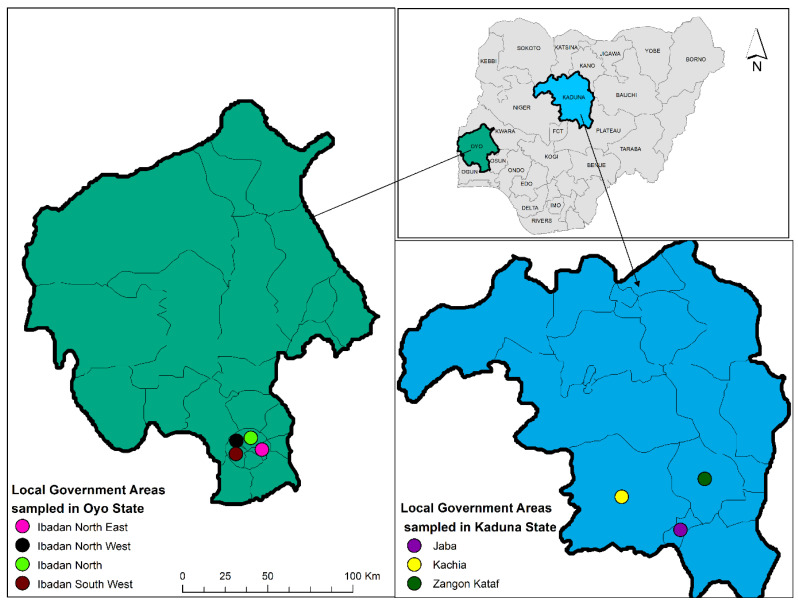
Map of the Nigeria showing locations in Kaduna and Oyo States where samples were collected.

**Figure 2:**
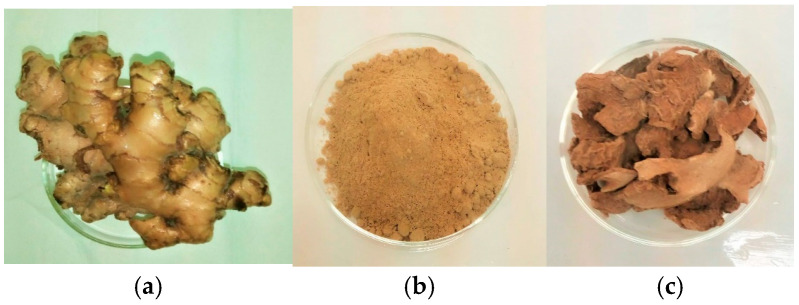
Ginger samples obtained in (**a**) fresh (**b**) milled (**c**) split-dried forms.

Samples were collected from major ginger-producing areas in Nigeria—Kaduna State (Jaba, Kachia and Zango local governments (LGs): GPS coordinates 9.4747461, 8.0124917; 9.8570726, 7.6885857; and 9.8374975, 8.4065268, respectively), at various points along the value chain (i.e., production, processing, marketing, and consumption) [11]. Samples were collected from all stages except consumption. Samples were also obtained from a major ginger-consuming area in Nigeria—Oyo State (Ibadan North, Ibadan Northeast, Ibadan Northwest, and Ibadan Southwest LGs: GPS coordinates 7.4477245, 3.8967116; 7.3939066, 3.9273483; 7.403651, 3.871402; and 7.3733705, 3.8589668) at the market stage (Figure 1). Ginger samples were collected in freshly harvested (fresh), split-dried, and milled forms (Figure 2). Data on sampling point, drying method and the storage containers used were collected for Kaduna-sourced samples. In Oyo State, data was collected on sampling point and storage container used. Samples were transported to the Pathology/Mycotoxin laboratory of the International Institute of Tropical Agriculture (IITA) and stored in the cold room at 4 °C until time for mycotoxin analysis.

### 2.2. Aflatoxin Analyses of Ginger and Inoculated Maize Samples

Aflatoxins were extracted from ginger samples using the method described by others [32,52] with modifications. Briefly, 10 g samples of each sample were blended with 100 mL of 80% methanol and 2 g of sodium chloride for 3 min and poured into Erlenmeyer flasks. Thereafter, the mixture in the covered Erlenmeyer flasks was agitated mechanically for 30 min on an orbit shaker at 400 rpm (Lab-line instrument, Inc., Melrose Park, IL, USA) to allow sufficient contact time between the extraction solvent and the substrate. Following this, the mixture was filtered through Whatman filter paper No 1. (Whatman Intl. Ltd., Maidstone, UK), and 20 mL of 10% sodium chloride solution and 12.5 mL of n-hexane were added to the filtrate and transferred to a separating funnel. Each separating funnel was shaken for one minute manually and left to stand and separate into phases. The lower phase was dispensed and combined in a separating flask with 10 mL of dichloromethane and again allowed to separate. Thereafter, the lower phase was dispensed into a bed of 20 g anhydrous sodium sulphate. This step was repeated with 7.5 mL of dichloromethane, and the obtained extract was left to evaporate to dryness in a fume hood overnight. The extracted aflatoxins from the inoculated maize samples were used to ascertain the aflatoxigenicity of the isolates, as previously described by Banwo et al. (2023) [50]. In brief, the extract was combined with 2 mL of dichloromethane in 1.5 mL Eppendorf tubes to form a reconstituted mix, that was homogenised with a vortex mixer (VWR, Digital Vortex Mixer, USA). Using clean capillary tubes. Calibrated TLC plates (silica gel 60, EMD Millipore, Darmstadt, Germany) were spotted with four microliters (4 µL) of aflatoxin pre-mix standards (Biopure, Romer Labs, Tulln, Austria) and 4 µL of each of the reconstituted sample extracts. The spotted plates were then developed in a solution of diethyl ether, methanol and distilled water (96:3:1, *v*/*v*/*v*) for 15 min and left to air-dry. Aflatoxin concentrations were quantified on the TLC plates using a densitometer (CAMAG TLC scanner 4) and Vision CATS version 3.1 software, (Camag AG, Muttenz, Switzerland).

### 2.3. Analyses of Heavy Metals in Sampled Ginger

For fresh ginger samples, they were first sliced into small pieces using a stainless-steel knife and oven-dried at 80 °C for 24 h to steady weight (Precision Scientific Oven, Model 18EM, Chicago, IL, USA). On the other hand, split-dried ginger and milled samples were not further dried. Un-milled samples were ground using a Warring Commercial blender (Torrington, CT, USA) before digestion. Heavy metal analysis was conducted at the One Health Platform, IITA Cotonou, Benin.

### 2.4. Sample Digestion and Analyses for Heavy Metals

The oven-dried ginger samples were sieved through a 2 mm mesh sieve for the extraction of heavy metals as described by [53], with modifications. Briefly, one gram of ginger powder was dissolved into 18 mL of deionized water in a Pyrex beaker, then combined with 10 mL of HNO_3_ and HCl (ratio 3:1). The mixture was left to stand overnight and then heated to 95 °C until a transparent solution was obtained.

Heavy metals such as copper (Cu), chromium (Cr), cadmium (Cd), lead (Pb), zinc (Zn), mercury (Hg) and manganese (Mn) were quantified using a Metalyser HM 3000 (Trace_2_O, Berkshire, UK). The quantification process was done by the standard addition method, which consisted of three phases according to the manufacturer’s instructions: (1) the conditioning of electrode, which was specific for each metal; (2) sample analysis using 70 mL extract per sample and requiring 5 min incubation; and (3) display of the results, including a corresponding graph on a multimedia tablet connected to the Metalyzer.

In this study the recovery was done to ensure reliability, samples were spiked by standard solutions of each targeted metal. Three concentrations were used in this case: 10 µg/kg, 20 µg/kg and 50 µg/kg. The samples were spiked with solutions of each metal, and the same sample was also tested without spiking (blank). They underwent the same extraction procedure as described above. The spiking was then carried out using the same method, with the amount added adjusted for each metal. A recovery rate was calculated with the following formula:

(1) $$\mathrm{Recovery}\;\mathrm{rate}=\frac{\mathrm{Spike}\;\mathrm{concentration}-\mathrm{blank}\;\mathrm{concentration}}{\mathrm{initial}\;\mathrm{concentration}}\times 100\%$$

a recovery ranging from 90 to 112% was obtained. This provided further confirmation of the technique used. The Lower Detection limit (LOD) of the device is 1 ppb and the Upper Quantification limit (LOQ) is 500 µg/kg. The specific LOD of Cu, Cd, Pb, Hg Mn is 1 µg/kg and 50 µg/kg for Cr and 5 µg/kg for Zn. The LOQ is 500 µg/kg for Cu, Cd, Pb, Hg, Zn, Cr and 200 µg/kg for Mn.

### 2.5. Health Risk Assessment of Toxicants in Ginger

The risk assessment of the analysed toxicants to Nigerian populations was calculated by estimating the risk of cancer for carcinogens (aflatoxins) and the risk of non-carcinogenic effects (for heavy metals). Information used to calculate these included the estimated probable daily intake and Margin of Exposure. The average body weight estimation was 60.7 kg, the average daily consumption of ginger used was 10 g, and the concentrations of the toxicants used was disaggregated by state, given that the toxicant concentrations from Oyo and Kaduna States were different. Estimations of cancer and non-cancer risk were calculated as described by [54,55].

Briefly, the Estimated Daily Intake (EDI) is projected from the product of concentration of toxicants (aflatoxin B1 or heavy metals) and average daily consumption (estimated at 10 g), divided by the average body weight (estimated at 60.7 kg). The Margin of Exposure (MoE) was calculated by dividing the benchmark dose lower confidence limit (BMDL) (with an extra cancer risk of 10% set at 170 ng/kg bw/day estimated by EFSA) [56]. The cancer slope factors (CSF) used for cadmium and lead were (0.38 mg/kg/day)^−1^ and (0.0085 mg/kg/day)^−1^, respectively [57]. The chance of developing cancer (carcinogenic risk [CR]) was estimated from the product of EDI and CSF for heavy metals (cadmium and lead). For exposure to aflatoxins, it was estimated as the product of EDI and average potency (AP). AP being calculated as 0.3 × proportion of Hepatitis B surface antigen (HBSAg)-positive prevalence rate + 0.01 3 × proportion of HBSAg-negative prevalence in Nigeria. The HBsAg-positive rate being 12.2% as reported from a national survey [58] and HBsAg-negative calculated as 87.8% (i.e., 100–12.2%).

Hazard Quotient (HQ) estimates the risk of non-carcinogenic effect (for the non-carcinogenic heavy metals) and is calculated as the ratio of exposure dose to reference dose, where a value below 1 indicates an exposure that is below the reference dose and unlikely negative health effect; whereas, values above 1 indicate a likely negative health effect [59].

Target Hazard Quotient is calculated as the product of the exposure frequency 350 days/year), exposure duration (30 years), ingestion rate of ginger (10 g/day) and heavy metal concentration (mg/kg) divided by the product of oral reference dose (mg/kg/day), body weight (kg) and average exposure time for non-carcinogens 365 days/year × 30 years), then multiplied by the unit conversion factor of 10^−3^.

Total THQ is the sum of the THQ for each of the toxicants.

### 2.6. Decontamination of Aflatoxin-Spiked Ginger

#### Extraction of Aflatoxins from Inoculated Maize Grains

Total aflatoxins were extracted from maize grains (five grams) inoculated with toxigenic *A. flavus* La3228, obtained from the IITA Pathology/Mycotoxin Laboratory. This strain, originally obtained from the Derived Savannah region in Nigeria, is a highly toxigenic *Aspergillus* strain [60]. This strain was inoculated on maize at a spore concentration of 500 μL of 1 × 10^6^ spores/mL. Following a seven-day incubation period, the inoculated grains were extracted for aflatoxin and quantified as previously described [61]. To achieve spiking concentration, aflatoxin extract was serially diluted with dichloromethane, concentrations confirmed by HPTLC, and then refrigerated at 4 °C until needed.

### 2.7. Obtaining Spiked Ginger at 50 ng/g

Ginger mash was spiked using aflatoxin extracts to concentration of 50 ng/g. The following formula was used to calculate the aflatoxin extract volume to be inoculated on ginger:

$$\mathrm{Aflatoxin}\;\mathrm{extract}\;\mathrm{volume}\;(\mathrm{mL})=\frac{\mathrm{final}\;\mathrm{sample}\;\mathrm{weight}\;(g)\times \mathrm{final}\;\mathrm{concentration}\;(\mathrm{ng}/g)}{\mathrm{concentration}\;\mathrm{of}\;\mathrm{extract}\;(\mathrm{ng}/g)}$$

### 2.8. Source of Lactobacilli and Preparation for Treatment

Two strains of lactobacilli were obtained from American Type Culture Collection (ATCC): *Lactiplantibacillus plantarum* ATCC 8014 (hereafter referred to as LP) and *Lactobacillus helveticus* ATCC 15009 (hereafter referred to as LH). The strains were cultured from glycerol suspensions on MRS broth, incubated at 30 °C for 48 h under anaerobic conditions, then streaked on MRS agar plates and incubated microaerophilically at 30 °C for 48 h. To obtain the desired concentration of lactobacilli for each of the strains, a loopful from the plates was transferred to 5 mL of sterile MRS broth, incubated for 48 h, then washed with PBS solution (pH 7.2) and centrifuged to obtain a clear cell suspension. Cell concentrations were determined using comparison with McFarland standards [62]. A concentration of 10^8^ live cells was used for decontamination experiments.

### 2.9. Treatment Groups for Decontamination Assessment

Treatment groups (comprising triplicates) were (i) un-spiked ginger alone (control), (ii) spiked ginger, (iii) spiked ginger with one of the two lactobacilli (iv) spiked ginger with both of the lactobacilli, (v) un-spiked ginger with lactobacilli. Milled ginger samples (10 g) previously quantified with concentrations <LOD of 2 ppb (as much as was practicable) were weighed into a sterile Falcon tube. Treatment groups were spiked to 50 ng/g and lactobacilli cells of 10^8^ live cells were used. Treatment groups were incubated at 30 °C for 24 h, 48 h, 72 h, 96 h and 120 h. Ginger was first autoclaved to remove extraneous organisms that could interfere with the assessment of the probiotic inclusion.

At the end of the incubation periods, experiments were terminated by extracting the samples for aflatoxins as earlier described. The extent of lactic acid bacteria-mediated aflatoxin decontamination was determined via recovery of aflatoxins from the samples as follows [50].

Recovery=(initial conc. − final conc.)/initial conc × 100%.

### 2.10. Descriptive Analysis and Analysis of Variance Among Variables

Descriptive analyses of the samples were conducted using Excel, analysis of variance (ANOVA) among variables was determined and means were separated using Student–Newman–Keuls test and box plots were developed using the R software (version 4.4.1).

## 3. Results

### 3.1. Concentrations of Aflatoxins and Heavy Metals Between Oyo and Kaduna States

Fifty-one (51) samples were obtained from Kaduna State and 44 samples from Oyo State. Samples from Kaduna State (a major ginger-producing state in Nigeria), were collected along the value chain, i.e., fresh ginger (five samples each), drying ginger (five samples each), ginger obtained from storage (five samples each) and ginger obtained from sales points (two samples each) at each of the local governments, totalling 18 fresh ginger, 33 split-dried samples. There were no milled samples obtained from Kaduna State. From Oyo State, samples were collected from markets: Ibadan North (nine samples), Ibadan Northeast (nine samples), Ibadan Northwest (17 samples) and Ibadan South-East (nine samples) local governments. These totalled 15 fresh samples, 14 split-dried and 15 milled samples.

**Figure 3:**
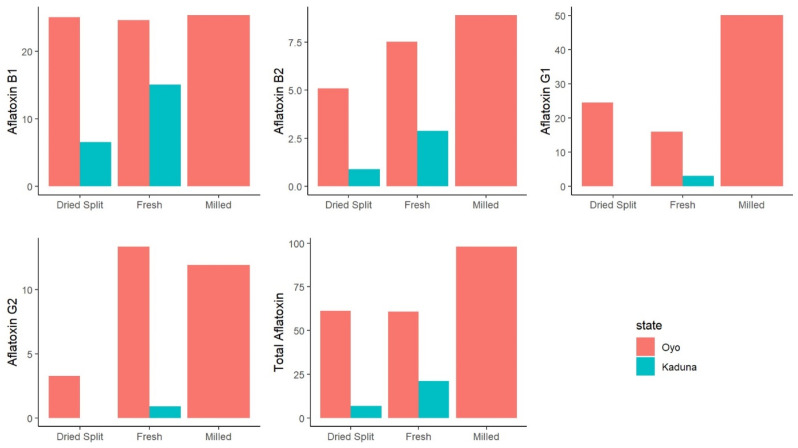
Aflatoxin concentrations in ginger sampled from Kaduna and Oyo States in different forms.

**Figure 4:**
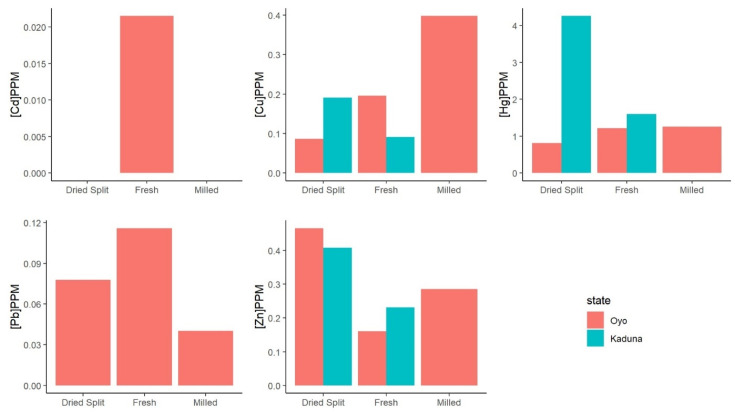
Heavy metal concentrations in ginger sampled from Kaduna and Oyo States in different forms.

Samples from Kaduna (50%) and Oyo (100%) States contained aflatoxins B1, B2, G1 and G2. Generally, aflatoxin concentrations were higher (*p* < 0.05) in Oyo State (73.63 ng/g) compared to Kaduna State (12 ng/g) (Table 1 and Figure 3). Overall, fresh and split-dried samples from Kaduna were less contaminated (*p* < 0.05) than fresh and split-dried samples from Oyo State (Table 2). Aflatoxins B1, B2 and G1 were higher in all milled samples compared to fresh and split-dried samples (Table 3). Cadmium (Cd), mercury (Hg), lead (Pb), copper (Cu) and zinc (Zn) were detectable in some of the samples (Figure 4). There was no lead or cadmium detected in ginger samples taken from Kaduna State.

Fifty percent (50%) of Kaduna samples were contaminated with aflatoxins. Aflatoxin concentrations ranged from undetectable concentrations to 82.2 ng/g. Aflatoxin concentrations in fresh samples ranged from 0.0 to 37.6 ng/g in Jaba, and 0.0 to 82.2 ng/g in Zango and were below detection levels in Kachia local governments. Whereas in split-dried samples the total aflatoxin concentrations were 0.0–19.0 ng/g in Jaba, 0.0–18.6 ng/g in Kachia, and 0.0–20.8 ng/g in Zango local governments.

Due to the low degrees of freedom for statistical analyses, descriptive analysis was conducted, for comparing samples among sampling locations, instead of ANOVA, as was done between states: in Jaba and Zango, more than 50% of the fresh samples contained above 10 ng/g samples (83% and 60% in Jaba and Zango respectively). However, 60% of the split-dried samples obtained from Zango contained above 10 ng/g total aflatoxins, but 27% (from Jaba) and 18% (from Kachia), respectively, contained above 10 ng/g total aflatoxins (Table 2). Total aflatoxin concentrations ranged from 7.7 to 184.7 ng/g in samples from Oyo State (Table 3). In all cases, except in Ibadan North, milled samples contained higher concentrations of aflatoxins compared to fresh and split-dried samples. The average total aflatoxin concentration in all the samples exceeded 10 ng/g. 

Cadmium and chromium were not detected in any of ginger samples. Among the fresh ginger samples, 100% and 86% from the Kaduna and Oyo States, respectively, met the safety requirements for lead set by the EC of 0.8 mg/kg. However, 51% of samples (Kaduna and Oyo States) did not meet the 0.1 mg/kg regulatory limit for set for mercury (Hg) in salt and supplements. There are no specific requirements for Hg in ginger, but as ginger is used as a dietary supplement [63,64] this standard may apply. All (100%) and 93% of ginger samples from the Kaduna and Oyo States, respectively, met the safety requirement of cadmium set by the European Commission (0.05 mg/kg). The guidelines for copper (Cu) and zinc (Zn) in adults are estimated at 5 mg/day for copper and 7 to 10 mg/day for zinc [65]. The concentrations of copper (maximum 2 mg/kg and 4 mg/kg in the Kaduna and Oyo States, respectively) and zinc (maximum 4 mg/kg and 3 mg/kg in the Kaduna and Oyo States, respectively) detected were within the guidelines set for these heavy metals (Figure 4). 

### 3.2. Storage Containers

In Kaduna, 100% of the fresh samples were stored in polythene sacks (high-density polyethylene (HDPE sacks); whereas, 31/33 (94%) of split-dried samples were stored in HDPE sacks and 2/33 (0.06%) were stored in bowls. Majority of the split-dried ginger (97%) samples were dried on bare surfaces, including bare cement or bare soil. Only one sample collected from the drying location (0.03%) was reported to be dried using both a solar dryer and the surface covered with tarpaulin.

In Oyo State, 100% of the split-dried samples were stored in polythene bags (low density polyethylene (LDPE) bags). However, fresh samples were stored in varied containers: raffia baskets (26.67%), LDPE polythene bags (26.67%), 4/15 HDPE polythene sacks (26.67%) and bowls (20%).

### 3.3. Health Risk Assessment of Toxicants in Ginger

#### 3.3.1. Risk Associated with Aflatoxin Ingestion in Contaminated Ginger

The average concentrations of aflatoxin B1 in the Oyo and Kaduna States were 25.1 ng/g and 9.0 ng/g, respectively. These resulted in a probable daily intake of 4.1 μg/kg bw/day and 1.5 µg/kg bw/day, respectively (at an estimated 10 g use per day)—Table 4. The risk of liver cancer (hepatocellular carcinoma) from exposure to aflatoxins in ginger in the Oyo State was three times higher than in the Kaduna State, at an annual risk of 18.7 cases/100,000, compared to 6.7 cases/100,000 persons/ year in Kaduna (MoE being 41.2 in the Oyo State, and 115.3 in the Kaduna State were below 10,000 in both states, indicative of adverse effects from dietary exposure [66]. The risk of developing cancer from lead and cadmium dietary exposure from both ginger sampled from the Kaduna and Oyo States was nil (0.000).

#### 3.3.2. Risk Associated with Heavy Metal Ingestion in Contaminated Ginger

The Estimated Daily Intake of mercury was within the limits for mercury in Oyo State but exceeded the limits for samples collected from Kaduna State by 60%. However, the estimated probable daily intake for the other heavy metals and aflatoxin B1 was within tolerable limits. On the contrary, for exposure to hazards in samples taken from Oyo State, lead was detected (there is no tolerable daily intake for lead) as this is considered harmful even in very minute concentrations. The probable daily intake of aflatoxin B1 was also 51% higher in samples collected from Oyo State. However, the daily intake of mercury, copper and zinc were within the tolerable limits.

The likely adverse effects from the consumption of these heavy metals were greater than one for mercury and lead, indicating that these are risk factors in the ginger that require management. The total THQ was 8.694 for samples taken from Oyo State and 5.482 for samples taken from Kaduna State. These values being above one indicates a risk that non-carcinogenic effects could occur from the ingestion of ginger from these states. The THQ was above one for mercury (1.727 and 5.481 for samples collected from Oyo and Kaduna States, respectively). THQ was 6.966 for lead for samples collected from Oyo State and below one for samples collected from Kaduna state. The THQ is calculated for as a cumulative risk over time. Therefore, although daily intake is within acceptable limits for mercury, a consistent accumulation can affect organs adversely and constitute a risk. There were no risks estimated (values were below one) for exposure to cadmium, copper and zinc in both the Oyo and Kaduna States. 

### 3.4. Aflatoxin Concentrations in Probiotic Study

Spiked ginger treated with LH or LP resulted in reduced aflatoxin concentration (49.5%) at the end of the 120 h incubation period. Total aflatoxin concentrations reduced similarly from 50 ng/g to 24 ng/g (after 120 h) when LP and LH were combined for spiked ginger treatment. However, there were difference between LH and LP treatments at the 72 h: whereas there was a steady reduction in aflatoxin concentrations when LP was used, when LH was used or combined with LP, at 72 h, the concentration returned to the original aflatoxin concentration before again reducing at 96 h and 120 h (Table 5).

## 4. Discussion

Ginger is widely consumed globally and is used as a supplement and an adjuvant in foods [52,63,64,67,68]. Its safety is therefore paramount as it could serve as a source of contamination for foods meant for human consumption. With the growing demand for natural supplements and the use of nature-based products, ginger use in diets has grown. This was particularly observed during the coronavirus disease pandemic (COVID-19) where the use of ginger was promoted for relieving symptoms and facilitating recovery, attributed to its antiviral and anti-inflammatory properties, among others [3,69].

The results of food safety attributes of ginger sourced from the Kaduna State (ginger-producing state) and the Oyo State (ginger-consuming state) show that toxicant contamination is present in the ginger value chain in the study locations. From results, contamination was detected at harvest, during drying, in storage and in the markets, indicating that ginger contamination can begin from the field. This necessitates the need to control contamination from production stage. Technologies such as the use of biological control have been utilised for pre-harvest aflatoxin control in cereals, legumes and spices [61,70]. As rhizomes develop underground, the ginger rhizomes can be easily associated with microorganisms present in the soil, such as *Aspergillus* species which includes mycotoxin-producing species and strains. Management of ginger at this stage using technologies like biological control products, such as Aflasafe and *Trichoderma* species, can offer protection to the crop [60,71]. However, this has not been reported in efficacy trials.

Aflatoxin concentrations were higher in the Oyo State compared to the Kaduna State. Kachia LG contained lower aflatoxin concentrations along the value chain, indicating locational differences with aflatoxin contamination in the sub-region. In Kaduna, elevated levels of ginger were recorded particularly at the sales points, indicating that accumulation of the toxin occurs between the time of harvest and its point of sale in the markets. Establishing pre-harvest protection can also proffer post-harvest benefits when the associated strains are non-toxin producers [72]. It is therefore critical for aflatoxin management of ginger to continue post-harvest, even where strategies have been implemented during pre-harvest to limit the contamination.

As part of post-harvest management, storage is an important component. From our results, HDPE sacks were used more frequently in the Kaduna State (100% of fresh ginger samples and 94% of split-dried ginger samples) than in the Oyo State (27% of fresh ginger samples and 0% of split-dried ginger samples). In the Oyo State, LDPE bags were used more frequently for storage (100% of split-dried ginger). This may have relevance to aflatoxin accumulation as HDPE have been reported to be better storage containers for ginger (resulting in lesser aflatoxin accumulation) than jute bags [73]. This aligns with our studies where samples taken from the Kaduna State had less aflatoxin concentrations than samples from the Oyo State (Table 1). Similarly, a comparison between HDPE films and Polyethylene Terephthalate (PET) bottles, rated HDPE as preferable to PET containers for storage of ginger (Collins Muga et al., 2019 [37]). Although, previously reported studies did not compare ginger stored in HDPE sacks with LDPE bags, which are more commonly used in the current study locations, they provide some insights into the utility of HDPE sacks for ginger storage as a preferred storage container. The additional factor of humidity could have contributed to higher aflatoxin accumulation in the Oyo State (being in the more humid rainforest agroecological region) historically has a higher relative humidity annually than Kaduna State (in the less humid Savanna agroecological region) [74].

Logistics and storage play crucial roles in post-harvest management, particularly in regions that are low-producing and high consuming areas. Ginger production occurs primarily in Kaduna; therefore, ginger in Oyo likely originated from Southern Kaduna and was transported to Ibadan, about 800 km away. Given the deficits in road infrastructure in Nigeria and the limited cold chain availability, the movement of food commodities leads to losses in quality and quantity. These can result in biosynthesis of aflatoxins in transit and storage by associated aflatoxigenic fungi; although, incidence of aflatoxigenic fungi is not reported in the current study. Additionally, Ibadan is generally more humid than Kaduna, providing more ideal conditions for aflatoxin biosynthesis post-harvest.

The risks of non-carcinogenic effects from all the heavy metals analysed as estimated from the THQ indicate that in both the Oyo and Kaduna States consumers are at risk from being affected from long term exposure to heavy metals in the ginger, as the THQ values are greater than one. However, for the risk of carcinogenic health effects from consumption of the sampled ginger, mercury exposure was a risk factor in both states; whereas, lead exposure constituted a risk in Oyo State, but Kaduna samples did not have detectable lead concentrations. It is important to understand the possible routes of contamination to control exposure of heavy metals in ginger along the value chain. Furthermore, understanding the incidence of heavy-metal related conditions such as neurological, allergic and infantile conditions (non-carcinogenic effects) and carcinogenic effects [41,42,43,44,45,46,47] within the study areas can provide some information that may be relevant for disease epidemiology among groups within the study location.

Lead exposure could be from industrial pollution to the soils, from holding containers used for packaging and handling of ginger along the value chain, leaching into water from lead pipes or lead-containing equipment. It is not clear the specific or primary routes of exposure through which lead has contaminated the ginger samples in this case. Consequently, this requires further investigation [75].

Based on the results obtained from this current study, open-air sun drying, particularly on bare soil or on cemented surfaces, was the dominant form of drying used. Only four (7.8%) of the samples collected were from covered surfaces in the Kaduna State (all from Zango local government). This indicates that sun-drying on uncovered surfaces is the common means of drying split ginger samples. Alternative solar drying innovations such as the parabolic solar dryers, offer superior results to open-air dryers—offering 65% less drying time [76,77]. Open-air drying on uncovered surfaces would predispose the ginger to microbes in the soil and from the environment. As *Aspergillus* species are ubiquitous, it can be a route of exposure to contamination leading to the accumulation of aflatoxins in the crop. Rapid drying is preferable as it would limit exposure to mycotoxins and reduce the rate of biosynthesis of aflatoxins [17].

Co-occurrence of heavy metals and aflatoxins is reported in these samples. Umar and Salihu, (2014) [78] reported the presence of non-essential and essential heavy metals in ginger, with higher concentrations in processed samples compared to the fresh samples. Among the samples obtained from Kaduna, all samples but one (a split-dried sample from Zango local government) contained detectable concentrations of at least one of the heavy metals investigated. The copper and zinc concentrations were below the toxicity levels for these metals, indicating that they were within the safe limits. On the other hand, 14% of the samples taken from markets in Oyo State contained unsafe concentrations of lead, and 51% of the samples from both Kaduna and Oyo States contained concentrations of mercury that exceeded the safe limits. These metals are naturally occurring and could have contaminated the rhizomes during production and post-harvest, as they are detectable in both fresh and processed samples. The incidence of these heavy metals is a subject of concern.

Due to the limited infrastructure, including poor road organisation, sub-optimal processing methods used along the ginger value chain by small holders, micro, small, and medium scale enterprises (MSMEs), the exposure of ginger to contamination is likely via the practices utilised post-harvest. It is therefore important for interventions to be in place to reduce the incidence of mycotoxins and ameliorate the risks associated with dietary exposure to aflatoxins. Although the milled ginger is reported to have a higher economic value than dried and fresh ginger [11], in the current study, milled ginger from Oyo State was reported as the most contaminated with aflatoxins. For this reason, there have been fora, including initiatives by Deutsche Gesellschaft für Internationale Zusammenarbeit (GIZ) GmbH, a German development agency, to improve the ginger value chain deficits in Nigeria [79]; export rejections and repatriations negatively impact incomes and the livelihoods of farmers and value chain players. Moreover, this undesirably also impacts the health and well-being of domestic consumers who are exposed to the repatriated ginger or the domestically traded ginger that is not tightly monitored and regulated for safety and quality in domestic value chains.

Efforts to control mycotoxins and other toxicants are therefore important for the food security (including food safety) of consumers.

The health risk assessment relied on empirical contamination data from this preliminary study; therefore, the inherent uncertainty in the selected analytical methods is captured in the calculations of the Hazard Quotient (HQ), Target Hazard Quotient (THQ), and carcinogenic risk estimates. Consequently, these risk predictions should be interpreted with this methodological limitation in mind.

The use of probiotics provided some value in the reduction in total aflatoxin concentration over the 120 h treatment period. Where there are slight increases above the spiked concentrations observed, this is possibly due to physical artefacts caused by minor losses of liquid (as the tubes were not repeatedly shaken to reintroduce the solvent) during the incubators cooling and heating cycles. Within the first 24 h, the aflatoxin concentration was the same as the starting concentration or with only marginal reduction. Reduction was more marked and consistent till the 120 h time point with *L. plantarum,* but not so when *L. helveticus* was present. Rather in the presence of *L. helveticus* (either alone or in combination with *L. plantarum*), concentrations reverted to their starting concentration at the 72 h time point. The authors surmise that the binding of the aflatoxins to the cells was temporarily reversed at 72 h due to a saturation of the binding sites of *L. helveticus* and possible crosstalk between the two LAB when *L. plantarum* was present [80,81]. Aflatoxin decontamination by lactobacilli has been described to occur by binding via the cell wall constituents, comprising mainly the peptidoglycan layer in addition to polysaccharides, proteins, and teichoic acids [59,82]. The parity between *L. helveticus* and *L. plantarum* at the 72 h incubation may occur because of differences in the architecture or biochemical processes during aflatoxin binding or saturation on the cell wall constituents. However, this has not been described and requires further investigation. Whereas the structural architecture of *L. helveticus* has been reported to include a cell wall thickness of 20 to 80 nm [83], that for *L. plantarum* has not been reported.

## 5. Conclusions

In this study, we find that multiple toxicants including aflatoxins and heavy metals co-occur in samples obtained from ginger. These were identified along the value chain and pose both liver cancer risks (for aflatoxins) and non-carcinogenic risks (for lead and mercury). There are location variations with respect to the extent of contamination and the risks of population exposure, requiring further investigation and context-specific management measures for consumer health protection. Probiotics can offer a means of decontamination of aflatoxin-contaminated ginger. However, more insights are needed to understand binding mechanisms and potentials for intermittent release of the toxins.

## Figures and Tables

**Table 1:** Average aflatoxin concentration in Kaduna and Oyo States.

	Average Aflatoxin Concentration in ng/g			
State	All Samples	Dried Split Samples	Fresh Samples	Percentage of Total Samples Above 10 ng/g
Kaduna	12.0 b	6.8 b	21.3 b	42
Oyo	73.6 a	61.2 a	79.4 a	98

ANOVA (α = 0.05) was used to compare means and were separated using Student–Newman–Keuls test. Letters that are different in the columns were statistically different from each other.

**Table 2:** Total aflatoxin concentrations of samples collected along the ginger value chain in Kaduna State, Nigeria.

Local Govts.	Stage of Sampling	N	Average Total Aflatoxin Concentrations in ng/g				Samples Above 10 ng/g (%)
			Harvest	Drying	Storage	Market	
Jaba	Split-dried	11	NA	7.1	0.6	14.6	27
	Fresh	6	23.5	NA	NA	42.4	83
Kachia	Split-dried	11	NA	8.7	0.0	0.0	18
	Fresh	6	0.0	NA	NA	13.5	17
Zango	Split-dried	11	NA	6.8	13.6	31.6	60
	Fresh	6	38.0	NA	NA	19.3	67

NA means Not Applicable.

**Table 3:** Total aflatoxin concentrations of samples collected from markets in Oyo State, Nigeria.

			Average Total Aflatoxin Concentration (ng/g)		Samples Above 10 ng/g (%)
Local Govts.	Ginger Form	N	Market	Range	
Ibadan North	Split-dried	3	14.9	7.7–20.2	67
	Fresh	3	45.2	42.5–50.2	100
	Milled	3	137.4	88.3–184.7	100
Ibadan Northeast	Split-dried	3	94.8	85.0–106.2	100
	Fresh	3	52.8	34.5–85.2	100
	Milled	3	64.7	43.3–85.6	100
Ibadan Northwest	Split-dried	5	82.3	20.1–123.7	100
	Fresh	6	79.6	39.4–109.3	100
	Milled	6	96.3	47.1–180.6	100
Ibadan Southwest	Split-dried	3	38.7	21.0–52.8	100
	Fresh	3	46.6	34.5–55.4	100
	Milled	3	95.8	73.7–119.9	100

**Table 4:** Estimated probable daily intake of heavy metals and aflatoxins in ginger.

Food Hazard	Estimated Probable Daily Intake (mg/kg bw/day)		Tolerable Intake (mg/kg bw/day)
	Oyo State	Kaduna State	
Mercury	0.180	0.572	0.23
Lead	0.007	0	no established TDI
Copper	0.037	0	0.04
Zinc	0.025	0.059	0.3
	Estimated probable daily intake (μg/kg bw/day)		PMTDI * (μg/kg bw/day)
Aflatoxin B1	4.1 μg/kg bw/day	1.5 μg/kg bw/day	2
Margin of Exposure	41.2	115.3	

* PMTDI = provisional maximum tolerable daily intake (this applies to aflatoxin B1). Estimated probable daily intake = EDI.

**Table 5:** Detoxification of aflatoxin-spiked ginger samples treated with *Lactiplantibacillus plantarum* and *Lactobacillus helveticus*.

Incubation Period (h)	Aflatoxin Concentrations (ng/g)		
	LP-Treated Spiked Ginger	LH-Treated Spiked Ginger	LP and LH Treated Spiked Ginger
0	50.0	50.0	50.0
24	42.75	36.18	50.83
48	32.57	31.67	36.12
72	21.29	54.71	41.07
96	27.30	25.90	28.87
120	24.11	23.61	24.07
Reduction (%)	48.22	47.22	48.14

LP: *Lactiplantibacillus plantarum*, LH: *Lactobacillus helveticus*.

## Data Availability

The original contributions presented in the study are included in the article, further inquiries can be directed to the corresponding authors.
